# User Perceptions of Virtual Hospital Apps in China: Systematic Search

**DOI:** 10.2196/19487

**Published:** 2020-08-12

**Authors:** Yuan Wang, Yuqiao Liu, Yancui Shi, Yanjun Yu, Jucheng Yang

**Affiliations:** 1 College of Artificial Intelligence Tianjin University of Science and Technology Tianjin China

**Keywords:** mobile apps, mHealth, remote consultation, China, app review analysis, user intent

## Abstract

**Background:**

Virtual hospital apps are mobile apps that offer functionalities of online consultation, medical guidance, health community forums, referrals, outpatient appointments or virtual hospital-to-home care services. With an increasing number of online medical and health care consulting services, virtual hospital apps have made health care more accessible and fairer for all, especially in China. However, they have occurred without control or regulation. User evaluation can provide directions to help apps optimize identification, lower risks, and guarantee service quality.

**Objective:**

We aimed to conduct a systematic search for virtual hospital apps in China. To get a global view, virtual hospital apps were assessed and characterized by means of quantitative analysis. To get a local view, we conducted a content feedback analysis to explore user requirements, expectations, and preferences.

**Methods:**

A search was conducted of the most popular Apple and Android app stores in China. We characterized and verified virtual hospital apps and grouped apps according to quantification analysis. We then crawled apps and paid attention to corresponding reviews to incorporate users’ involvement, and then performed aspect-based content labeling and analysis using an inductive approach.

**Results:**

A total of 239 apps were identified in the virtual hospital app markets in China, and 2686 informative corresponding reviews were analyzed. The evidence showed that usefulness and ease of use were vital facts for engagement. Users were likely to trust a consulting service with a high number of downloads. Furthermore, users expected frequently used apps with more optimization to improve virtual service. We characterized apps according to 4 key features: (1) app functionalities, including online doctor consultation, in-app purchases, tailored education, and community forums; (2) security and privacy, including user data management and user privacy; (3) health management, including health tracking, reminders, and notifications; and (4) technical aspects, including user interface and equipment connection.

**Conclusions:**

Virtual hospitals relying on the mobile internet are growing rapidly. A large number of virtual hospital apps are available and accessible to a growing number of people. Evidence from this systematic search can help various types of virtual hospital models enhance virtual health care experiences, go beyond offline hospitals, and continuously meet the needs of individual end users.

## Introduction

Public health focuses on improving and protecting health and well-being among large groups of people. However, the borders of time and space limit equality in countries’ medical systems, especially in rural areas and for vulnerable groups [1,2]. With the rapid development of the internet, virtual hospitals can provide registration, remote medical advice, and online doctor consultations. Researchers suggest that 25% to 70% of health care solution seeking does not need a face-to-face appointment with a doctor [3]. Importantly, the Chinese government is vigorously promoting this new type of health service to support health care reform [4,5]. Virtual hospitals play an effective role in reinforcing traditional health care and allow for a flexible relationship between physicians and patients [6] in many countries around the world [7-9].

Currently, China is the largest and fastest-growing market for the mobile internet. The number of mobile internet users in China is 1.31 billion and the mobile data traffic reaches 110.1 billion GB [10]. Residents in China have grown accustomed to using mobile health services for health-related information and health solution retrieval [11,12]. Existing virtual hospital apps in China mainly focus on the functionalities of online consultation, medical guidance, health community forum development, referral, or outpatient appointments. Recently, some apps have started providing appointments for virtual hospital-to-home care services [13]. Due to fair access opportunity to high-quality doctors on virtual hospital apps, the majority of people are willing to pay more for the service [14]. Some researchers have studied online consultation from doctors’ perspectives [9].

However, few researchers focus on user evaluation of Chinese virtual hospital apps. There is little research on how user engagement happens and why users abandon a virtual hospital app. To the best of our knowledge, user reviews and basic app data are accessible and valuable [15,16], but have not been fully explored, especially for virtual hospital apps. Hence, we conducted a systematic search using statistical analysis and aspect-based content analysis to review apps of this theme, determine user engagement, and discuss user needs, expectations, concerns, and preferences for virtual hospital apps. We hope the in-depth analysis can provide guidance on the further optimization of apps and their services.

## Methods

### Selection of App Markets

According to the 2018-2019 China Mobile App Store Market Annual Monitoring Report from iiMedia Research, 360 Mobile Assistant was the most popular Android app market in China [17]. Hence, we selected 360 Mobile Assistant as the typical Chinese Android app market and the App Store as the typical iOS app market to retrieve virtual hospital apps.

### Identification of Virtual Hospital Apps

On November 18, 2019, we crawled mobile apps in 360 Mobile Assistant (Android) and App Store (iOS). We used the following 7 Chinese keywords to conduct a preliminary search in app markets: see a doctor, medication guide, consultation, hospital registration, doctor, medication, medical treatment. We selected the apps that appeared in both Android markets and iOS markets for in-depth analysis. We found that many apps were short-lived, similar to mental health apps [18,19]. More than half of the studied apps were revised roughly every four months [18]. Due to these observations, we excluded the new apps that were first released after July 2019. For these apps, the information was insufficient and unstable for objective analysis. Three investigators screened the names and descriptions of these apps and then tested functionalities of the apps to distinguish between virtual hospital–related apps and unrelated apps. We excluded the apps that more than one investigator labeled as not relevant to the theme. In this way, we identified the included virtual hospital apps. We downloaded and crawled the qualified apps with the researchers’ related information.

### Apps for Analysis

**Figure 1:**
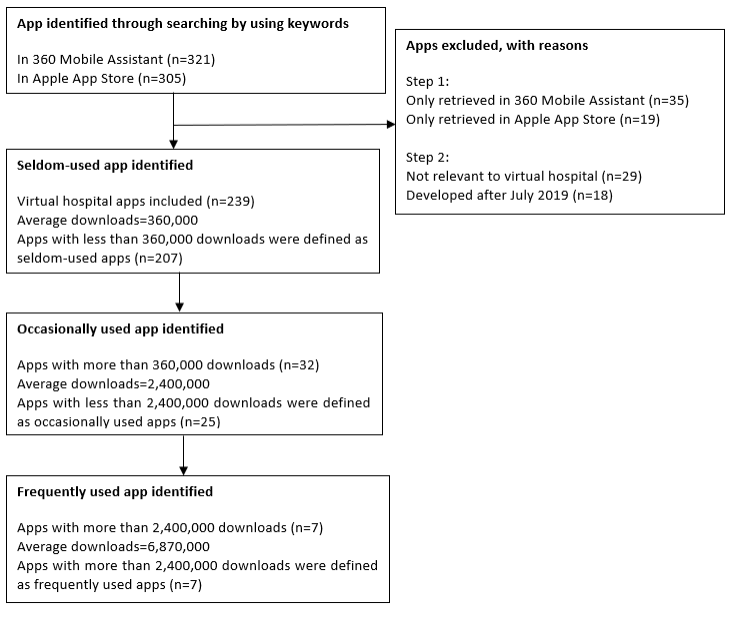
Flowchart of app classification selection processes.

All of the apps were sorted by number of downloads. For each included app, extracted data consisted of (1) flat user score, reflecting perceived satisfaction of a user; (2) the number of downloads, showing the measure of usage frequency and intention; (3) the number of positive, negative, and neutral ratings; (4) content of reviews; and (5) the last updated date of an app. Critically, due to the fact that Apple’s App Store requires a minimum number of reviews before releasing average ratings (ie, small numbers are suppressed), we think that the score on the App Store is more referential. Thus, we used average ratings in App Store. While App Store does not provide the number of downloads, we used the number of downloads from 360 Mobile Assistant for analysis. We divided apps into three categories according to the number of downloads: (1) frequently used apps, (2) occasionally used apps, and (3) seldom-used apps. First, apps with fewer than the average number of downloads of all determined apps were defined as seldom-used apps. Second, apps with fewer than the average number of downloads of the remaining apps were defined as occasionally used apps. Other apps were defined as frequently used apps. Procedures are shown in Figure 1.

### Aspect-Based Content Labeling of Valuable User Reviews

According to the proposal put forward by Mendiola et al [19] and review data, we sum up the following four aspects that concern users: (1) app functionalities, including online doctor consultation, in-app purchases, tailored education, and community forums; (2) security and privacy, including user data management and user privacy; (3) health management, including health tracking, reminders, and notifications; and (4) technical aspects, including user interface and equipment connection.

For qualified apps, we selected and reviewed user-generated reviews after the last update of the 7 most popular apps. We found 4630 reviews. Before aspect-based content analysis, we filtered poor-quality or fake reviews from our data set based on the following rules: (1) review expresses only positive or negative emotions and without mentioning any specific aspects, such as “good!” or ”very bad!“ (n=1902); and (2) review is an apparent case of human manipulation, such as advertising reviews that have nothing to do with the corresponding app (n=42).

**Figure 2:**
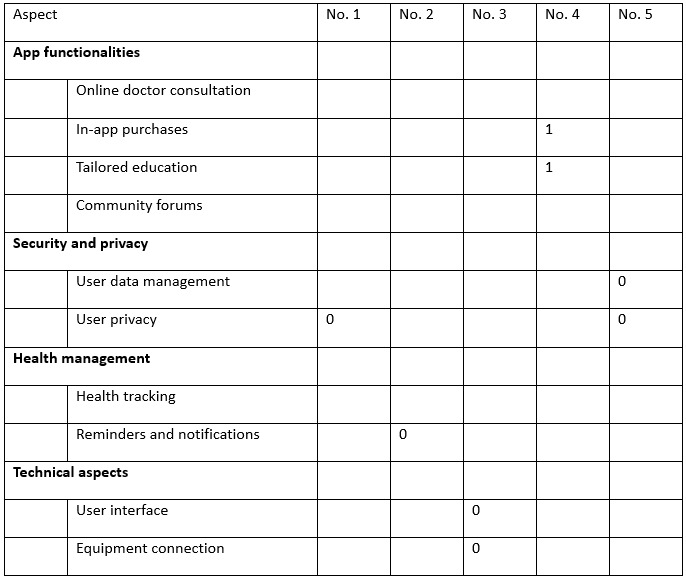
Aspect-based content labeling for the review examples in Textbox 1.

After screening, we got a clean review data set (n=2686), where each review was authentic and specific. On the clean review data set, we asked 3 investigators to use a binary system to assign ”1“ to a particular aspect in a review in which the user was satisfied or ”0“ to indicate that the user was dissatisfied with a particular aspect. Investigators independently conducted quality assessments and the final results were decided by majority vote. Examples are shown in Textbox 1 and Figure 2. Note that most of the reviews only included one or two aspects (2570 reviews with one aspect each, 116 reviews with two aspects each).

**Examples of reviews.**

**No. 1.** “This app always takes mandatory access to my address book data and leaks out my personal privacy!”

**No. 2.** “The sound of the reminder to take my medicine is too low and unattractive and there is so much noise at home that I often can't hear it!”

**No. 3.** “The interface design is too complicated! I hope they make the design a little simpler. In addition, the connection between bracket and app is always unstable, easy to disconnect.”

**No. 4.** “Every time the doctor can provide me with reasonable advice according to my physical condition and deliver medicine directly to the door. There are formal invoices and after-sales protection!”

**No. 5.** “I can neither upload my data to my Wechat, nor share it with my friends. I hope this feature is added!”

## Results

### App Characteristics

Table 1 summarizes the characteristics of included virtual hospital apps, including the statistical information of downloads and rating scores. According to the search strategy and data filtering, we took a total of 239 apps for analysis. The average rating score was 3.8 out of 5 and the average number of downloads was 36,464. We included a total of 92,366 rating scores, including 90,106 positive scores, 1512 negative scores, and 748 neutral scores. Note that people can give rating scores without reviews for apps. Thus, there was not a one-to-one correspondence between content of reviews and the numbers of ratings. In addition, apps had an average of 386 scores, including 377 positive scores, 3 neutral scores, and 6 negative scores per app.

The results in Table 1 show that there was no linear relationship between the number of rating scores and the number of downloads. Frequently used apps only accounted for 2.9% (7/239) of the number of virtual hospital apps but accounted for 63.06% (5,496,000/8,715,000) of all downloads. There was not a big difference in average rating scores between the frequently used apps and the occasionally used apps, but the seldom-used apps scored notably lower than the other two. For frequently used apps, negative rating scores accounted for the highest proportion of the total comparison theory, followed by seldom-used apps, and lastly occasionally used apps. People set higher requirements for popular virtual hospital apps (ie, frequently used apps).

**Table 1:** Characteristics of included virtual hospital apps.

Characteristic	Frequently used apps	Occasionally used apps	Seldom-used apps	All
Apps, n (%)	7 (2.93)	25 (10.46)	207 (86.61)	239 (100)
Downloads, n (%)	5,496,000 (63.06)	2,208,000 (25.34)	1,011,000 (11.60)	8,715,000 (100)
Positive ratings, n (%)	59,687 (97.17)	15,063 (98.49)	15,356 (98.15)	90,106 (97.55)
Neutral ratings, n (%)	577 (0.94)	83 (0.54)	88 (0.56)	748 (0.81)
Negative ratings, n (%)	1162 (1.89)	148 (0.97)	202 (1.29)	1512 (1.64)
Ratings, n (%)	61,426 (100)	15,294 (100)	15,646 (100)	92,366 (100)
Rating score, mean	4.8	4.6	3.7	3.8
Downloads, mean	785,142	88,320	4884	36,464
Positive ratings, mean	8526	602	74	377
Neutral ratings, mean	83	3	1	3
Negative ratings, mean	166	6	1	6
Number of ratings, mean	8775	611	75	386

### Case Study of Selected Apps

We ranked apps according to their average rating scores and the number of downloads in the frequently used apps, occasionally used apps, and seldom-used apps categories. We took the top 3 and the last 3 apps in each category for in-depth analysis.

The results are shown in Table 2. For frequently used apps, the difference between average rating scores was not large, but the number of downloads and the number of positive, neutral, and negative ratings varied greatly. Although Dr Chunyu had the most downloads (17.98 million), it also had the most neutral rating scores and received a low average ranking score in this category. The majority of apps with more downloads had more positive reviews, neutral reviews, and negative reviews. There are also good virtual hospital apps in the occasionally used and seldom-used app categories, for example Good Doctor for People and Dr Ruyi, with average rating scores of 4.9*.* For seldom-used apps, apps with higher average rating scores had more downloads than apps with lower scores, except Dr Thumb*.* We observed that some apps in the seldom-used apps category specialized in types of virtual hospitals, such as Dr Doodle for pregnant women and newborns and Dr Warmth for video consultation.

**Table 2:** Characteristics of the typical apps in the frequently used app, occasionally used app, and seldom-used app categories (ordered by average rating scores).

App (app’s name in Chinese Pinyin)		Score	Downloads (in millions)		Positive reviews, n (%)		Neutral reviews, n (%)		Negative reviews, n (%)
**Frequently used apps**									
	Considerate Doctor (Tie Xin Yi Sheng)	4.9		5.54		9254 (99.92)		3 (0.03)	4 (0.04)
	Online Family Doctor (Jia Ting Yi Sheng Zai Xian)	4.9		3.39		1088 (99.18)		5 (0.46)	4 (0.36)
	Ping An Good Doctor (Ping An Hao Yi Sheng)	4.8		15.13		29,445 (96.80)		258 (0.85)	715 (2.35)
	Enjoy Health (Yi Jia Jian Kang)	4.7		3.16		895 (95.93)		5 (0.54)	33 (3.54)
	Dr Chunyu (Chun Yu Yi Sheng)	4.7		17.98		11,197 (95.66)		243 (2.08)	265 (2.26)
	Online Good Doctor (Hao Dai Fu Zai Xian)	4.6		4.96		1944 (98.08)		10 (0.50)	28 (1.41)
**Occasionally used apps**									
	Good Doctor for People (Ren Min Hao Yi Sheng)	4.9		2.16		816 (99.51)		0 (0.00)	4 (0.49)
	9K Doctor (9k Yi Sheng)	4.9		1.01		401 (99.01)		0 (0.00)	4 (0.99)
	Dr Lighthouse (Deng Ta Yi Sheng)	4.9		0.63		1137 (99.91)		0 (0.00)	1 (0.09)
	39 Ask for Doctor (39 Wen Yi Sheng)	4.4		0.36		158 (86.81)		6 (3.30)	18 (9.89)
	Female Private Doctor (Nv Xing Si Ren Yi Sheng)	4.2		1.28		298 (86.63)		17 (4.94)	29 (8.43)
	Dr Almond (Xing Ren Yi Sheng)	3.8		0.40		21 (43.75)		3 (6.25)	24 (50)
**Seldom-used apps**									
	Dr Ruyi (Ru Yi Yi Sheng)	4.9		0.23		738 (99.73)		0 (0.00)	2 (0.27)
	Dr Doodle (Du Du Yi Sheng)	4.9		0.20		3163 (99.81)		2 (0.06)	4 (0.13)
	Dr Half (Ban Ge Yi Sheng)	4.9		0.18		382 (99.48)		1 (0.26)	1 (0.26)
	5U Family Doctor (5U Jia Ting Yi Sheng)	2.5		0.002		0 (0.00)		0 (0.00)	3 (100.00)
	Dr Warmth (Wen Nuan Yi Sheng)	3.0		0.002		0 (0.00)		2 (100.00)	0 (0.00)
	Dr Thumb (Mu Zhi Yi Sheng)	2.6		0.09		7 (46.67)		5 (33.33)	3 (20.00)

### Aspect-Based Content Analysis of User Reviews

We analyzed authentic and specific reviews, defined in the section “Aspect-Based Content Labeling of Valuable User Reviews.” The 4 themes were (1) app functionalities, (2) security and privacy, (3) health management, and (4) technical aspects. The themes and associated aspects are summarized in Table 3.

From Table 3, we observe statistical information regarding user concerns about virtual hospital apps. First, we compared the total number of reviews for each aspect. The majority of reviews expressed opinions about online doctor consultation, health tracking, and user data management for virtual hospital apps in China, while fewer reviews discussed community forums, user privacy, and tailored education. Second, we determined the ratio of positive and negative evaluations. Many reviews praised online health services (948/600, 91.3% for online doctor consultation; 148/152, 97.4% for reminders and notifications). About 80% (32/40) of reviews showed positive opinions about community forums within virtual hospital apps, indicating that users like understanding, sharing, and discussing health-related issues in apps. However, it is imperative to improve security and privacy in virtual hospital apps, especially for user data management, which 86.0% (394/458) of reviews complained about. Details of typical user opinions about each aspect are described below.

**Table 3:** Themes and aspects of virtual hospital apps.

Aspect		Total, n (%)^a^	Positive, n (%)	Negative, n (%)
**App functionalities**				
	Online doctor consultation	600 (22.34)	548 (91.33)	52 (8.67)
	In-app purchases	358 (13.33)	188 (52.51)	170 (47.49)
	Tailored education	80 (2.98)	44 (55.00)	36 (45.00)
	Community forums	40 (1.48)	32 (80.00)	8 (20.00)
**Security and privacy**				
	User data management	458 (17.05)	64 (13.97)	394 (86.03)
	User privacy	66 (2.46)	2 (3.03)	64 (96.97)
**Health management**				
	Health tracking	554 (20.63)	290 (52.35)	264 (47.65)
	Reminders and notifications	152 (5.66)	148 (97.37)	4 (2.63)
**Technical aspects**				
	User interface	286 (10.65)	190 (66.43)	96 (33.57)
	Equipment connection	208 (7.74)	10 (4.81)	198 (95.19)

^a^Percentage is out of 2686 ratings.

### Topic 1: App Functionalities

#### Online Doctor Consultation

Online doctor consultation, which has been adopted by all virtual hospital apps, is a functionality that provides convenient outpatient service online. This is the best feature for user engagement, with the highest number of positive reviews (n=548). For consulting, users preferred video chatting and shared more details with pictures of their condition. For example, a user said, “I am a patient with dermatosis. It is so painful that I can't describe how I feel, so I want to upload some videos or photos about my illness for help or advice.” For consulting quality, most users were satisfied due to the truthfulness of doctors’ information. Users particularly wanted to reach high-quality and certified doctors during web-based consultation services. A user said, “I hope apps can collect a large amount of information about doctors to provide valuable references for patients, including doctor’s reputation and experience.” With this information, regardless of time or location, patients can find and reach high-quality doctors from all over the country.

#### In-App Purchases

A total of 52.5% (188/358) of users mentioned that they felt it was convenient to buy drugs or other health care–related goods in apps. Users said that they were concerned about whether the drug provided was safe enough. Users further talked about their opinion that (1) apps should take actions to avoid counterfeit medications and (2) after-sales protection could make them feel at ease when buying drugs in virtual hospital apps. Related to online doctor consultation, users also wished that doctors who recommended medications were professional enough to judge disease symptoms and know side effects of drugs. Moreover, users wanted apps to show clear and accurate information about the terms and conditions of in-app purchases.

#### Tailored Education

Users desired apps to have the ability to tailor schemes for different populations by analyzing personal data. For patients with chronic diseases, users prefer personalized knowledge and consulting recommendations in apps based on their previous records of vital signs, laboratory test results, or medical records. For example, a patient with diabetes said, “This app scored for various histopathological indicators by my uploaded data, then returned my physical health condition to me and provided nutrition knowledge and counseling. I like it!”

#### Community Forums

Social features in virtual hospital apps were a significant influence on ratings. We found a total of 40 reviews about community forums, 80% (32/40) of which provided positive opinions. Users wished for apps to provide a forum to elaborate on particular health care topics, share arduous experiences, discuss their views, and even share treatments for diseases. Patients with chronic conditions wanted to have doctors join discussions to share knowledge and experience, ask for advice on diagnosis and treatment for specific patient cases, and discuss issues about their disease and related topics. Pregnant women preferred an in-app feature to chat with other pregnant women, just like chatting with each other when lining the hospital corridors.

### Topic 2: Security and Privacy

#### User Data Management

On the premise of ensuring data security, users wanted to get more health data administrative privileges. Reviews that discussed data management made up the highest number of negative reviews (n=394). Users wanted to synchronize personal health data between multiple apps or to migrate data to another app. Apps should provide intuitive and easy features to query, display, and download data. Meanwhile, apps with local data management should remind users to save data correctly. For example, a pregnant user said, “After re-installing the app, I lost all my data for five months of pregnancy, which made it impossible for me to continue my physical management.” In this case, data retention was unsatisfied.

#### User Privacy

For online inquiries about diseases, users required that their data be protected and anonymized. Users complained about privacy settings within the highest ratio of negative reviews (64/66, 97%,). They needed authentication, authorization, and a privacy mechanism to protect their data. For general diseases, some users were embarrassed to consult with their own identity. They requested the ability to have a doctor consultation in an anonymous way and have an option to destroy their data immediately after consultation. Users expected to be notified via messages or emails if their accounts were logged in abnormally, such as logging in with new devices. Those who were willing to consider sharing data also emphasized data control. They talked about the risk of sharing their health data. Some of them preferred local databases that were disconnected from the internet or distributed databases over several servers.

### Topic 3: Health Management

#### Health Tracking

Health tracking is the right way for users to be aware of changes in health status. In this way, it is essential to understand how apps collect user data and provide processed visualized data to users. Only 52.4% (290/554) of reviews were positive about health tracking in virtual hospital apps. These reviews mentioned that an automatic and effective way of health tracking in apps could help make doctor consultation better. For some patients with chronic conditions, app features that monitored daily blood pressure and heartbeat data were helpful. Patients with insomnia hoped that the app could access sleep data about sleep latency, sleep duration, sleep efficiency, and sleep quality assessment to provide to doctors during consultation.

#### Reminders and Notifications

Personalized reminders can reinforce behavioral changes. A total of 97.4% (148/152) reviews supported timely and accurate reminders and notifications. For negative reviews, users expressed challenges with limited alert customization, especially in terms of the alarm loudness. A user commented, “No matter what tone, the tone is too soft and doesn‘t ring long enough to wake me up.” Users mentioned that customization with a ringtone was “an essential feature since it is how you will be notified.” Sometimes, users hoped that notifications could not be closed forcibly. In addition, users said that apps should remind them of potential risks for improper usage or possible adverse effects along with the reminders and notifications.

### Topic 4: Technical Aspects

#### User Interface

For user interface design, users thought simplicity was the most important design feature and argued for adaptation for vulnerable groups and multiple modes. An excellent user interface can have a powerful impact on the usability and user experience of an app. About 66.4% (190/286) of reviews expressed satisfaction with the user interface of virtual hospital apps in China.

Users appreciated clear, intuitive, and helpful design apps that were easy to use (ie, navigation was inherent). Reviewers complimented apps that had a “clean and simple format” and a “beautiful layout.” On the contrary, reviews commented on “clunky” user interfaces. For example, apps should try to avoid lots of extra buttons and submenus. Elderly people in particular frequently listed inconvenience of use, which influenced their engagement. Moreover, the interface design should follow the same pattern. That is, all graphic elements (ie, typographies, icons, and buttons) should have a consistent appearance. The features of each component (ie, navigation menu, lists, and photo gallery) should be identified.

Besides these features, virtual hospital apps could pay more attention to health, for example, by providing a night mode for eyesight protection. We also found that monolingual apps sometimes made consultation difficult. These apps were difficult for foreigners to register for and use. Some users said that multilingual settings could benefit foreign user groups; even basic machine translation could help. These reviews demonstrate that medical service via virtual hospital apps in China could think about globalization to serve more people.

#### Equipment Connection

In China, health monitoring and wearable devices are becoming extremely popular [20], as they can record and transmit real-time, health-related data from a patient’s home to any medical situation. However, this aspect was a top complaint along with user privacy. The percentage of reviews about equipment connection that were negative was 95.2% (198/208). Users often complained that (1) apps could not detect the device, (2) apps could not connect to the device for data transmission, (3) data transferred to the client were missing or inaccurate, (4) there was an unstable connection, (5) there was poor compatibility when connecting another device, (6) they were unable to unpair, or (7) there were external interruptions (eg, incoming calls or messages). Besides these issues, users mentioned that both Wi-Fi and Bluetooth could be supported or that the app could be made to work in flight mode.

## Discussion

### Overview

This study explored user preferences and requirements for virtual hospital apps to predict and analyze current models and future directions of the medical service market with mobile internet. First, we provided a statistical description of full data to compare distributions of rating scores and number of downloads with 3 different categories. Second, we conducted in-depth aspect-based content analysis to discover opinion details about interest in and barriers to participation. In this study, the app and review selection strategy benefited research, with better generalization and time-efficiency. Despite many works that have discussed virtual hospitals from the perspective of economics [21] or doctors [9], this paper is the first to provide an in-depth report and highlight situations from the users’ perspective.

### Principal Findings

End user engagement and follow-up are prerequisites for sustained promotion and effectiveness. In the field of virtual hospitals, frequently used apps with the most downloads received the highest average rating scores (4.8/5), while there was great variation in the average rating scores among seldom-used apps. Interestingly, the ratios between positive and negative reviews were similar in frequently used, occasionally used, and seldom-used apps, although the Matthew effect was clearly seen in the number of downloads. We noticed that frequently used apps suffered from more negative reviews (1162/61,426, 1.89%) with detailed user experience. Users tending to give high rating scores for those popular apps had higher expectations for conversational features, personalization, performance, and high-quality content and preferred putting forward the related proposals. Frequently used apps should take responsibility for possible updates and improvements.

We noticed that high-probability access to individualized expert consultation was the main reason for both new and old user engagement. First, health care service apps can greatly relieve unequally distributed medical resources in China. Medical resources are geographically unbalanced, especially for rural areas and for persons with a disability [2]. For virtual hospitals in apps, users can access a virtual hospital regardless of time or location. All that is needed is an internet-enabled phone. In positive reviews, users said that they liked to try virtual hospital apps because they were a considerable distance away from good medical resources. When patients reported that they were cured by a doctor's treatment in an app, they were more likely to pay for in-app purchases for further health services [14]. Users felt comfortable with famous, professional doctors who also worked for offline hospitals rather than those who were from unknown or ambiguous sources. Our findings suggest that virtual hospital apps should rely on actual medical conditions and qualified physicians and medicines. With the support of government policies [4,5], users in China were willing to trust that apps could invite high-quality doctors to trade their leisure time for online consultation. Second, most users were satisfied with the prompt response, consulting attitude, and advanced knowledge of doctors in apps. Users saved waiting time, reduced transportation costs, and shared fair doctor navigation. Third, a multichannel communication strategy for points of patient engagement was welcomed. Besides video chatting, which is similar to an offline service, new consultation options in apps, such as reservation calls, voice messages, graphic messages, video messages, or plain text, could relieve patients’ tension and give patients thinking time.

Compared with online doctor consultations, users were wary of health products in in-app purchases, such as medicines, supplements, probiotic food, and home health care devices. About 47.5% (170/358) of users raised questions because distribution channels for medicine and medical devices lacked trusted verification. It was hard for customers to protect their rights with in-app purchases. Stakeholders need to pay more attention to consumers’ rights protection in the selling of health-related products.

Users cared about their health-related data. On the one hand, users sought convenient and flexible data usage, including data association, data synchronization, data transmission, and data sharing across multiple apps and external devices. Specifically, inferred from patients’ comments, we found that some users wanted to switch apps and share their data with other communities or doctors. However, that is a big challenge now [22]. An agreement is needed to put forward sharing and canonical management of health data in China. On the other hand, users asked for precision and integrity for data management. Accurate and complete health consultation records are often a great help to detect or review changes in physical and psychological indicators. Users wanted to learn from their health data history.

With regard to interactivity and visual settings, users preferred broad individualization. Personalization can enhance convenience and user-friendliness. For tailored education, relevant and meaningful individualized health knowledge provides engagement, serendipity, and high value to users, while irrelevant or unindividualized health care information displays make users feel unengaged. This type of individualization has been shown to be vital for sustained engagement [23]. For user interface, about 66.4% (190/286) of comments showed positive attitudes to the current design. The others, including foreign app users, elderly persons, and visually impaired users, asked for alternative customization view modes for promoting large-scale, easy usage. The design of indicative icons and feedback functions required more consideration. For reminders and notifications, most users found such functions helpful for consultation appointments, abnormal account access, or medicine plans. Notifications allow users to tackle health problems and to schedule time for maintaining health [24,25]. Personalized reminders can better reinforce behavioral changes in app users.

Community forums based on peer support, education for health practice, health promotion, and emotional support can serve as an effective supplement to professional doctor consultation. It can relieve China’s insufficient medical resources. A total of 1.48% (40/2686) of reviews mentioned that social health education is a powerful force in human health. People share their health experiences, knowledge, and practical support, and others learn from that. Such client-to-client communication and question answering could meet the needs of finding people who have similar health issues. In reviews, users provided tips to promote peer support, for example, placing forum joining in a prominent position during patients’ in-app journeys.

Equipment connection and user privacy remain a considerable challenge in virtual hospital apps. Satisfaction of these two aspects was relatively low, with a satisfaction rate of only 4.8% (10/208) and 3.0% (2/66), respectively. With fast growth of wearable devices [20], inexpensive health-related wearable devices provide everyone the possibility of constantly tracking personal health data. However, due to inaccurate data estimation and poor connection of devices, the user experience was negative. Inaccurate data estimation limits data usage, such as using data as an accurate and valid reference for doctor consultation. Data show that user privacy protection was not paid enough attention. Almost all comments about this issue were negative. Apps lacked explicit features to protect user accounts, especially for abnormal log-in attempts. Users suggested that email or a message alert could be an easy way to improve security. App makers need to consider how to balance user security and privacy with flexible data access.

Virtual hospital apps are convenient and suitable for two types of users: (1) users who are far away from good medical resources in time or space, especially for rural users and users with disabilities, and (2) users who require medical support and group support but are uncomfortable with face-to-face communication. However, users who demand high security and privacy need to practice caution during app selection and usage.

A practical finding from our analysis is that the Chinese market has begun to pay attention to privacy, security, personalization, and socialization in virtual hospital apps. We estimate that these will provide better solutions in future development.

### Limitations

This research has some limitations. First, we explored only 2 app markets, although they are the most representative ones for Android and iOS operating systems in China. Second, we were not sure about the recall ratio for the whole mobile virtual hospital market because there were other popular ways to access virtual hospital services with mobile internet, such as temporal mobile online meetings, Wechat applets, and online chat groups. Except for platform operators, data of these tools were incomplete and inaccessible in most situations. Thus, we mainly focused on public app markets. Third, we limited content analysis on frequently used apps, as we thought that other data may suffer from severe bias due to the small number of helpful reviews and downloads. However, it is known that this can be a risk for sampling strategies. Finally, this research did not include opinions or usage experiences of medical workers, such as doctors and nurses.

### Conclusions

Virtual hospital apps are growing in popularity with the high pace of mobile internet in China. User evaluation provides a valuable insight for app development and online medical service reform. This study provides mobile internet–based statistical data for medical service providers, domain policy makers, and potential related stakeholders, and it provides aspect-based content analysis to support a better understanding of virtual hospital app users.
